# Pre-Meal Effect of Whey Proteins on Metabolic Parameters in Subjects with and without Type 2 Diabetes: A Randomized, Crossover Trial

**DOI:** 10.3390/nu10020122

**Published:** 2018-01-25

**Authors:** Ann Bjørnshave, Jens Juul Holst, Kjeld Hermansen

**Affiliations:** 1 Department of Endocrinology and Internal Medicine, Aarhus University Hospital, DK-8000 Aarhus, Denmark; kjeld.hermansen@aarhus.rm.dk; 2 Danish Diabetes Academy, DK-5000 Odense, Denmark; 3 NNF Centre for Basic Metabolic Research, University of Copenhagen, DK-2200 Copenhagen, Denmark; jjholst@sund.ku.dk; 4 Department of Biomedical Sciences, University of Copenhagen, DK-2200 Copenhagen, Denmark

**Keywords:** pre-meal, whey proteins, type 2 diabetes, postprandial lipemia

## Abstract

Diabetic dyslipidemia with elevated postprandial triglyceride (TG) responses is characteristic in type 2 diabetes (T2D). Diet and meal timing can modify postprandial lipemia (PPL). The impact of a pre-meal of whey proteins (WP) on lipid metabolism is unidentified. We determined whether a WP pre-meal prior to a fat-rich meal influences TG and apolipoprotein B-48 (ApoB-48) responses differentially in patients with and without T2D. Two matched groups of 12 subjects with and without T2D accomplished an acute, randomized, cross-over trial. A pre-meal of WP (20 g) or water (control) was consumed 15 min before a fat-rich meal (supplemented with 20 g WP in case of water pre-meal). Postprandial responses were examined during a 360-min period. A WP pre-meal significantly increased postprandial concentrations of insulin (*P* < 0.0001), glucagon (*P* < 0.0001) and glucose-dependent insulinotropic peptide (GIP) (*P* < 0.0001) in subjects with and without T2D. We detected no effects of the WP pre-meal on TG, ApoB-48, or non-esterified fatty acids (NEFA) responses to the fat-rich meal in either group. Paracetamol absorption i.e., gastric emptying was delayed by the WP pre-meal (*P* = 0.039). In conclusion, the WP pre-meal induced similar hormone and lipid responses in subjects with and without T2D. Thus, the WP pre-meal enhanced insulin, glucagon and GIP responses but did not influence lipid or glucose responses. In addition, we demonstrated that a WP pre-meal reduced gastric emptying in both groups.

## 1. Introduction

Persons with type 2 diabetes (T2D) have a 2-3-fold increased risk of developing cardiovascular disease (CVD) [1,2]. Postprandial hypertriglyceridemia (PPL) [3] is part of the diabetic dyslipidemia that contributes to excess morbidity and mortality in T2D [2]. PPL with increased chylomicron levels, reflected by apolipoprotein B-48 (ApoB-48), is an independent risk factor for CVD in persons with and without T2D [4,5,6]. Following food consumption, lipolysis of triglycerides (TG) in chylomicrons generates TG-rich remnant lipoproteins that can accumulate in the arterial intima and lead to atherosclerosis [7]. 

The quality of both dietary protein and fat influence the magnitude of PPL in persons with and without T2D [8]. Thus, we demonstrated that whey proteins (WP) from milk acutely reduce PPL compared with other proteins sources in obese subjects without [9] and with T2D [10]. WP is a potent insulinotropic substance that lowers postprandial blood glucose responses to a carbohydrate-rich meal [11,12,13,14]. Interestingly, WP also beneficially influences blood glucose when consumed as a pre-meal before a carbohydrate-rich meal [12,13]. The beneficial impact of a WP pre-meal on blood glucose and insulin responses before a carbohydrate-rich meal has repeatedly been documented [15,16,17]. The mechanism of action of the WP pre-meal may be related to the mixture of amino acids (isoleucine, leucine, valine, lysine and threonine), which are abundant in WP and trigger a pronounced early postprandial insulin response and a decrease in glucose [18].

The metabolic impact of a WP pre-meal before a fat-rich meal has not previously been studied in T2D. We know that the PPL is exaggerated in T2D [3] and that WP is a potent insulinotropic substance [11,12,13,14]. Therefore, we hypothesized that a WP pre-meal before a fat-rich meal reduces TG and ApoB-48 responses more pronouncedly in subjects with T2D than in subjects without T2D. We also suggested that WP as a pre-meal is more advantageous in this respect than as part of the main meal. We tested our hypothesis by evaluating postprandial responses of TG, ApoB-48, non-esterified fatty acids (NEFA), insulin, glucagon, glucose, glucagon-like peptide-1 (GLP-1), glucose-dependent insulinotropic peptide (GIP) and S-paracetamol and by assessing appetite regulation (using a visual analogue scale (VAS)).

## 2. Materials and Methods

### 2.1. Design

The present study was performed as an acute, randomized, cross-over study. A pre-meal of WP (20 g) or water (control) was consumed 15 min before a fat-rich meal (which was supplemented with 20 g WP after the water pre-meal). The test days were separated by a washout period of approximately one week. Subjects were randomly allocated into the sequence of pre-meals by use of coin flip. The day prior to each test day, the participants consumed a standard diet handed out to them. The energy intake was 7000 kJ for women and 9000 kJ for men. The composition of the diet was 54 energy percent (E%) from carbohydrates, 20E% from proteins and 26E% from fat (fat composition: saturated fatty acids (SFA): 23.2%, monounsaturated fatty acids (MUFA): 44.6% and polyunsaturated fatty acids (PUFA): 21.7%). Participants were instructed to avoid alcohol, painkillers containing S-paracetamol and demanding physical activity the day before the tests. 

After 12 h overnight fasting, the participants showed up in the morning in our clinic (Aarhus University Hospital, Aarhus, Denmark). A catheter was placed into a cubital vein for blood sampling. Thereafter, we collected anthropometric data and fasting blood samples (for plasma and serum) and urine. The participants also completed the first set of visual analogue scale (VAS) questions. VAS was applied to assess satiety, hunger, fullness and prospective consumption. For VAS, an iPad Air (Apple, Silicon Valley, CA, USA) was used with the app ‘VAS’ version 1.02 (University of Basel, Basel, Switzerland). The participants were presented to one question at a time. They had to range their answer on a line expressing the most positive and negative rating possible at each end. Participants were not allowed to discuss the answers with each other. Additionally, the VAS was completed immediately before the fat-rich main meal and then every 30 min in the postprandial period after the standardized meal.

### 2.2. Subjects

Thirty subjects were recruited from February to March 2015 through local newspapers and from the Outpatient Clinic at the Department of Endocrinology and Internal Medicine at Aarhus University Hospital, Denmark. The inclusion criteria were: ≥18 years and weight stability for at least three months for both groups. T2D was defined by glycated haemoglobin (HbA1c) ≥ 48 mmol/mol and non-diabetics by HbA1c < 48 mmol/mol. All interested subjects were screened. They were excluded in case of clinically significant CVD, renal or endocrine diseases, alcohol, or drug abuse; and if they were being treated with steroids, were pregnant, breast feeding, or had a significant psychiatric medical history. Regular medication was accepted if the dose had been stable for a minimum of 4 weeks and was unchanged throughout the study period. For the T2D group, treatment with dipeptidyl peptidase 4 (DPP-4) inhibitors and GLP-1 receptor agonists was not accepted. The subjects did not receive payment.

Twenty-four Caucasian subjects—12 non-diabetics and 12 T2D patients—were divided into two groups and included in the present study. The groups were matched for sex, age and BMI. The study was conducted at the Department of Endocrinology and Internal Medicine, Aarhus University Hospital, Denmark, from March to April 2015. In the T2D group, 7 were treated with lipid-lowering drugs, 12 with antidiabetics (Metformin) and 8 with antihypertensive drugs. In the non-diabetic group, 1 was treated with a lipid-lowering drug and 3 with antihypertensive drugs. All subjects provided their written informed consent. The protocol was approved by the Central Denmark Region Committees on Health Research Ethics (1-10-72-368-14) and was conducted in accordance with the Declaration of Helsinki. The present study was registered with ClinicalTrials.gov (ID: NCT02343471).

### 2.3. Interventions

The pre-meal consisted either 20 g of WP (LACPRODAN DI-9224 INSTANT, Arla Foods Ingredients Group P/S, Viby J, Denmark) dissolved in 200 mL water or a drink of 200 mL water (control). 20 g WP has an energy content of 311 kJ and contains 17.6 g protein, 0.3 g fat and <0.02 g lactose. The pre-meals were ingested at the time point −15 min within 2 min. Blood samples were drawn at the time point −10 and 0 min. A standardized, fat-rich breakfast meal was served at the time point 0 min and consumed within 15 min. The test meal consisted of white bread and rye bread, butter, salami, cheese, milk, egg, bacon and decaffeinated coffee. The energy distribution (17E% from protein, 15E% from carbohydrates and 68E% from fat (fat composition: SFA: 47.8%, MUFA: 27.8% and PUFA 6.5%)) was calculated based on the product labels. When the pre-meal was included in the main meal it was ingested during the meal. The total energy content was 3900 kJ. In addition, the standardized meal was supplemented with 20 g WP in case of water pre-meal and water in case of WP pre-meal. The participants also received 1.5 g paracetamol (3 × 500 mg tablets of Pinex^®^, Actavis, Hafnarfjordur, Iceland) and an additional 100 mL water. Blood samples were drawn at 15, 30, 60, 90, 120, 180, 240 and 360 min. During the entire test period, the participants were allowed to drink 200 ml tap water.

### 2.4. Blood Analysis

Serum (ApoB-48 and S-paracetamol) was separated from full blood after centrifugation at 2000× *g* for 15 min at room temperature. Plasma was immediately separated by centrifugation at 2000× *g* for 15 min at 4 °C. Plasma samples were taken in ethylene-diamine-tetra acetic acid (EDTA) (TG, NEFA, glucose, insulin, GLP-1 and GIP) and aprotonin (glucagon). Both serum and plasma samples were frozen at −20 °C and stored at −80 °C. ApoB48 was analysed from serum by an enzyme-linked immunosorbent assay (ELISA) assay (Code B/3645, Shibayagi Co. Ltd., Shibukawa, Japan) [19]. TG, NEFA, glucose and S-paracetamol were analysed by COBAS c111 system (Roche Diagnostics Gmbh, Manheim, Germany). The following methods and commercial kits were applied: for plasma TG and NEFA, an enzymatic colorimetric method (ref. no. 04657594190 for TG (Roche Diagnostics Gmbh, Manheim, Germany) and ref. no. 434-91795 and 436-91995 for NEFA (Wako Chemicals Gmbh, Neuss, Germany)). Plasma glucose was analysed by an enzymatic reference method with hexokinase (ref. no. 04657527190 (Roche Diagnostics Gmbh, Manheim, Germany)). Serum S-paracetamol was quantified by a colorimetrical method with arylacylamidease (ref. no. 03255379 190 (Roche Diagnostics Gmbh, Manheim, Germany)). Plasma insulin was analysed with an enzyme immunoassay (Code no. K6219; Dako Denmark A/S, Glostrup, Denmark). Glucagon was measured using a radioimmunoassay kit specific for pancreatic glucagon with glucagon antibody and ^125^I-glucagon (Cat. No. GL-32K; Millipore, St. Charles, MO, USA). Total GLP-1 and GIP concentrations were measured using a radioimmunoassay. Plasma samples were prepared by extraction with 70% ethanol (vol/vol, final concentration). An antiserum specific for C-terminal in GIP (code no. 867) was used. The sum of intact GLP-1 and its primary metabolite (=total GLP-1) was measured using an antiserum specific for the C-terminal of GLP-1 (no. 89390) [20].

### 2.5. Statistical Analysis and Calculations

The power calculation was based on previous results from our group [10] on the primary effect parameter of postprandial TG response incremental area under the curve (iAUC). A total of 12 subjects were needed in each group to detect a 20% difference between the two groups at a statistical power of 80% and a significance level of *P* < 0.05 (α = 0.05; 1 − β = 0.8). The anticipated dropout rate was set to 20%. Clinical characteristics and results are given as means with a 95% confidence interval (CI), unless otherwise stated. All statistical calculations were performed in STATA 14.2 (StataCorp LP, College Station, TX, USA) and graphical elements were accomplished in GraphPad Prism 7 (GraphPad Software, San Diego, CA, USA). ANOVA for repeated measurements was used to examine the effect of diabetes, intervention and time on the postprandial responses using subject and day as random variables; BMI and age as covariates; and gender, visit, randomization and lipid-lowering drugs as systematic variables. Baseline was also used as a covariant for glucagon, glucose and GLP-1, due to significant difference in fasting values. If no second- or third-order interaction between diabetes, intervention and time was found, the main effects of these factors were tested. Differences between individual time points were analysed only if there was second-order interaction. Model validation was accomplished by reviewing the probability plots of the residuals. A log-transformation was performed if the assumption about normally distributed data not was met (evaluated by quantile-quantile plots). *P*-Values < 0.05 were considered statistically significant.

## 3. Results

A total of 24 subjects—12 with T2D and 12 without diabetes—completed the study. Table 1 presents the participants’ baseline characteristics. As expected fasting plasma glucose concentration (*P* < 0.0001) and HbA1c (*P* < 0.0001) were significantly higher in the T2D than in the non-diabetes group. Three subjects withdrew after randomization for reasons unrelated to the study. Table 2 shows postprandial concentrations to selected time points and interacting effects between diabetes, intervention (WP as a pre-meal) and time. We observed no third-order interaction effects between diabetes, intervention and time for any of the measured biochemical parameters, except for glucagon. Likewise, we observed no interaction between diabetes and intervention for any of the parameters. 

### 3.1. Triglycerides, ApoB-48 and NEFA

**Figure 1:**
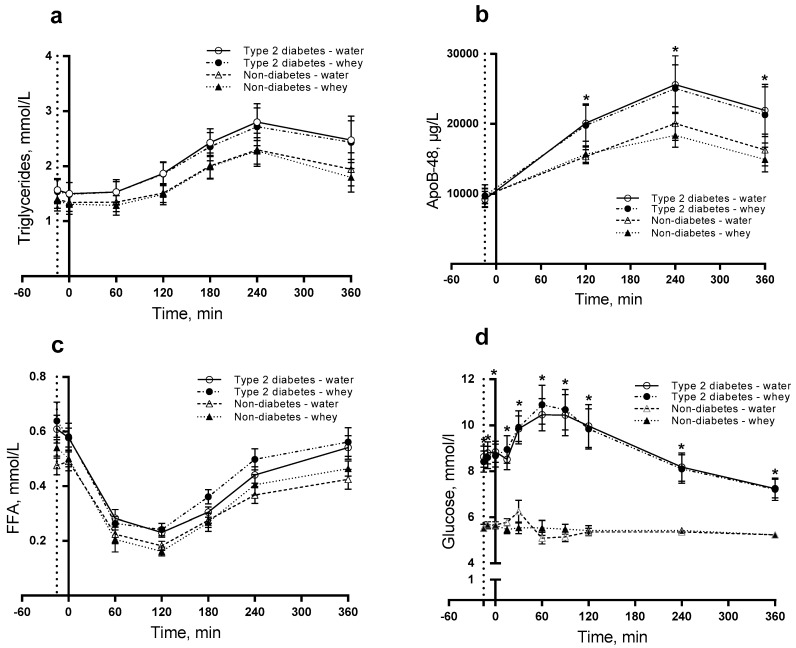
Postprandial responses of triglycerides (TG) (**a**); ApoB-48 (**b**); non-esterified fatty acids (NEFA) (**c**) and glucose (**d**). 12 T2D and 12 non-diabetic subjects were observed after consumption of 20 g whey proteins (WP) or water as a pre-meal ingested 15 min prior to a fat-rich meal. Dotted vertical line (y = −15) indicates pre-meal consumption. Data are given as mean ± standard error of the mean (SEM), *n* = 24. * indicates differences between subjects with and without type 2 diabetes. ANOVA for repeated measurements was used to examine the effect of diabetes, intervention and time on the postprandial responses.

For TG, we observed no difference in postprandial responses and thus no effects of diabetes, intervention, or time (Figure 1a). For ApoB-48, we observed a significantly higher postprandial concentration in subjects with than without T2D (*P* = 0.0039) (Figure 1b). The ApoB-48 concentration was significantly higher for the T2D group than for non-diabetics after 120 min (*P* = 0.025), 240 min (*P* = 0.012) and 360 min (*P* = 0.016). For NEFA, we observed no difference in postprandial responses (Figure 1c), thus we looked for individual main effects. We observed that diabetic subjects had a significantly increased NEFA concentration (*P* = 0.0040) independently of time and intervention. 

### 3.2. Insulin, Glucagon and Glucose

**Figure 2:**
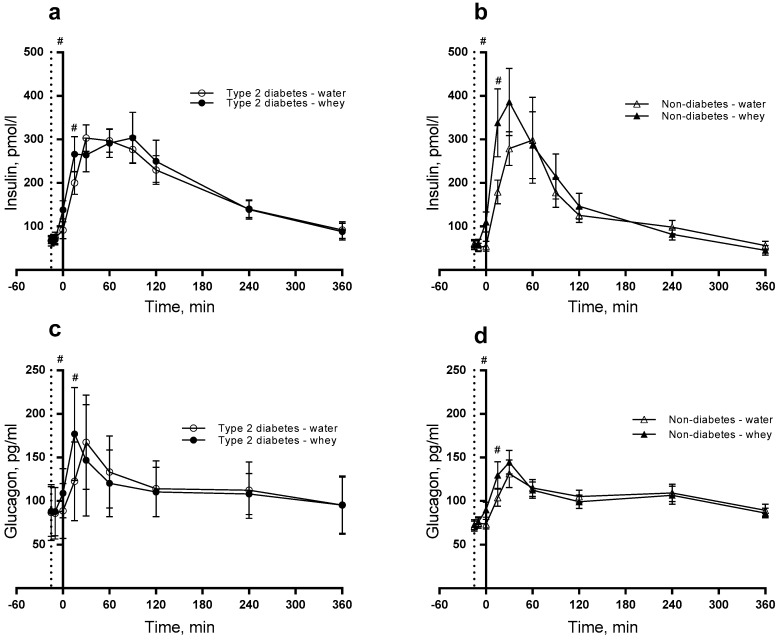
Postprandial responses of insulin (**a**,**b**) and glucagon (**c**,**d**). 12 T2D (**a**,**c**) and 12 non-diabetic subjects (**b**,**d**) were observed after consumption of 20 g whey proteins (WP) or water taken as a pre-meal 15 min prior to a fat-rich meal. Dotted vertical line (y = −15) indicates pre-meal consumption. Data are presented as mean ± SEM, *n* = 24. ^#^ indicates differences between pre-meals (WP and water). ANOVA for repeated measurements was used to examine the effect of diabetes, intervention and time on the postprandial responses.

Postprandial insulin concentration significantly increased during the postprandial period in T2D group compared with non-diabetics (*P* < 0.0001). Likewise, the intervention (WP pre-meal) caused higher postprandial insulin concentration than water (*P* < 0.0001) during the time period (Figure 2a,b). The WP pre-meal caused larger insulin concentration at time point 0 min (*P* = 0.002) and 15 min (*P* = 0.047) than the control. The presence of diabetes resulted in a significantly higher insulin concentration after 360 min (*P* = 0.048). For glucagon, we observed a difference in postprandial responses induced by diabetes, intervention and time (*P* = 0.0340) (Figure 2c,d). Additionally, we observed a significantly higher postprandial glucagon concentration after WP than water (*P* < 0.001) with higher concentrations at time points 0 (*P* = 0.021) and 15 min (*P* = 0.001). As seen in Table 2, we found postprandial glucose concentration higher in T2D subjects than in non-diabetic subjects (*P* < 0.0001), being significant at all time points (Figure 1d). The effect of the pre-meal was similar in the two groups. 

### 3.3. GIP and GLP-1

**Figure 3:**
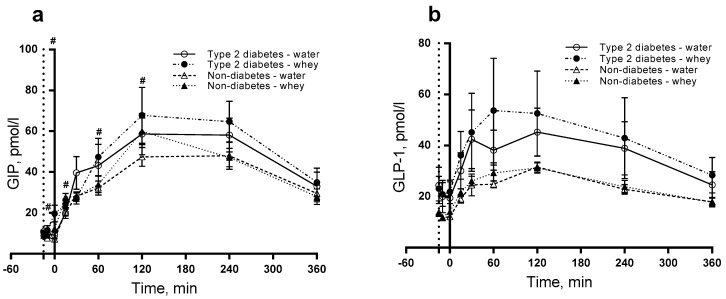
Postprandial responses for glucose-dependent insulinotropic peptide (GIP) (**a**) and glucagon-like peptide 1 (GLP-1) (**b**). 12 T2D and 12 non-diabetic subjects were observed after consumption of 20 g whey proteins (WP) or water as a pre-meal 15 min prior to a fat-rich meal. Dotted vertical line (y = −15) indicates pre-meal consumption. Data are presented as mean ± SEM, *n* = 24. ^#^ indicates differences between pre-meals (WP and water). ANOVA for repeated measurements was used to examine the effect of diabetes, intervention and time on the postprandial responses.

Figure 3a shows a significant effect on GIP of the WP pre-meal with a postprandial concentration higher than water (*P* < 0.0001) with larger GIP concentrations at time points −10 (*P* = 0.033), 0 (*P* < 0.0001), 15 (*P* = 0.001), 60 min (*P* = 0.046) and 120 min (*P* = 0.022). For GLP-1, we observed no difference in postprandial responses (Figure 3b).

### 3.4. S-Paracetamol

**Figure 4:**
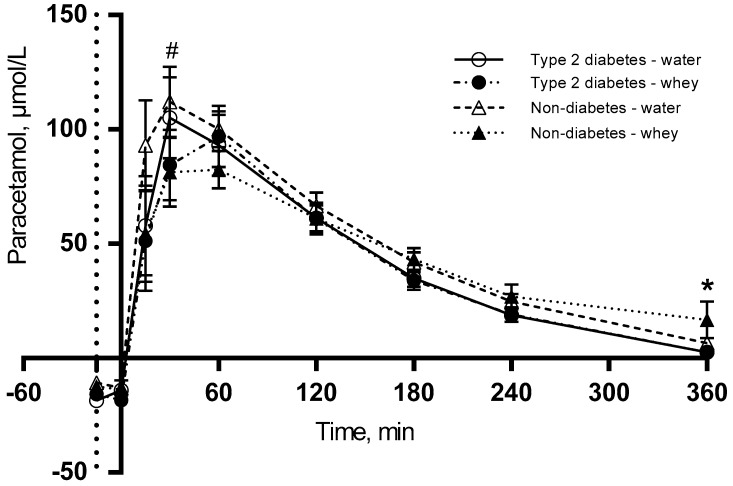
Postprandial responses for S-paracetamol. 12 T2D and 12 non-diabetic subjects were observed after consumption of 20 g whey proteins (WP) or water as a pre-meal 15 min prior to a fat-rich meal in. Dotted vertical line (y = −15) indicates pre-meal consumption. Data are presented as mean ± SEM, *n* = 24. * indicates differences between subjects with and without type 2 diabetes. ^#^ indicates differences between pre-meals (WP and water). ANOVA for repeated measurements was used to examine the effect of diabetes, intervention and time on the postprandial response.

Figure 4 shows that WP pre-meal delays gastric emptying in both groups and S-paracetamol peaks to the time point 60 min, whereas the water pre-meal peaks 30 min postprandially. No S-paracetamol was detected in the fasting state. The postprandial concentration of S-paracetamol was significantly lower after 30 min for WP than for water (*P* = 0.003). Additionally, we observe a significant difference between the two groups (*P* = 0.0271), with a lower S-paracetamol concentration after 360 min in the T2D group (*P* = 0.039) than in the non-diabetes group. 

### 3.5. Subjective Appetite

We found no second-order interactions for the subjective appetite parameters of fullness (*P* = 0.7859), satiety (*P* = 0.8530), hunger (*P* = 0.9778) or prospective consumption (*P* = 0.8482). However, there was a main effect of intervention (*P* = 0.0135) on the appetite parameter of fullness, indicating that a WP pre-meal reduces the fullness feeling. Diabetes had a main effect on prospective consumption, reducing the appetite parameter (*P* = 0.0216) (Supplemental Table S1). 

## 4. Discussion

In the present study, we investigated the impact of a WP pre-meal consumed prior to a fat-rich meal on responses of TG and ApoB-48 in subjects with and without T2D. Furthermore, we tested whether a 20 g WP pre-meal had a more pronounced effect on lipid responses than 20 g WP being part of the fat-rich meal. We have chosen a 15-min pre-meal interval due to the fact that diabetic subjects often take some of their medicine 15–20 min before their meals. Interestingly, we found a differential effect on hormone (insulin, glucagon and GIP) and lipid responses. The responses of the three hormones were initially elevated by a 20 g WP pre-meal in both groups compared with the 20 g WP being part of the main meal. The responses of ApoB-48 were higher in T2D than in non-diabetic subjects. However, the WP pre-meal did not influence TG, ApoB-48, or NEFA responses during the postprandial period in either of the two groups. We observed that a WP pre-meal delayed gastric emptying more than WP as part of the main meal in both groups. 

We have previously studied the effect of WP on postprandial lipid metabolism in subjects with T2D [10]. In that study 45 g WP was co-ingested with a fat-rich meal opposed to the present study where we tested the impact of a pre-meal of 20 g WP ingested 15 min prior to a fat-rich meal. Contrary to our previous results, we found no impact of a 20 g WP pre-meal on the circulating TG concentrations. The lack of influence of 20 g WP in the present study may be due to the lower amount of protein. However, we have chosen this amount WP since previous studies in healthy subjects have demonstrated physiologic effects of pre-meals containing 9–20 g WP on glucose concentrations [16] and gastric emptying [17].As expected, the postprandial concentration of ApoB-48 was significantly higher in subjects with than without T2D. Whether this difference is related to a higher production, a reduced clearance or both of chylomicrons was not clarified in the present study. However, it has previously been demonstrated that there is an intestinal ApoB-48 overproduction in insulin-resistant humans [21]; likewise, there is an absence of an acute inhibitory effect of insulin on chylomicron production in T2D [22]. In line with this, we found that the ApoB-48 iAUC was significantly higher in T2D than in non-diabetic controls (data not shown). Also, the NEFA response (tAUC) was significantly higher in the T2D than in the non-diabetic group, which may be due to insulin resistance and impaired ability to suppress NEFA postprandially (data not shown).

S-paracetamol was used as a marker of gastric emptying. As expected no S-paracetamol was detected at baseline, reflecting that the subjects were not taking paracetamol before the test. Interestingly, we observed that S-paracetamol responses were influenced by the WP pre-meal. After 30 min, the S-paracetamol concentration was significantly lower in both groups after the WP pre-meal than when WP was part of the main meal. This indicates that the gastric emptying was delayed by the WP pre-meal in both subjects with and without T2D, potentially because the pre-meal introduces a prolonged intestinal exposure to WP. It has previously been demonstrated that WP can delay gastric emptying in obese non-diabetic [23] and T2D subjects [12]. Since hyperglycaemia reduces gastric emptying [24], we expected a more pronounced delay in T2D than in non-diabetics. However, our study did not detect a difference between the two groups. One reason may be that the mechanism slowing gastric emptying in healthy subjects involves GLP-1 in a negative feedback loop. But in subjects with T2D, this feedback loop may be disturbed due to abnormally delayed gastric emptying, which is caused e.g., by hyperglycaemia [24]. Since we did not observe a WP-induced increase in GLP-1 in the present study this cannot explain a delayed gastric emptying. An alternative mechanism may involve a direct or indirect effect of the amino acids being released from WP. A third explanation could be the length of the intervention. Potentially long-term intervention will display this effect. Recently, it was demonstrated that the delay of gastric emptying remains after 4 weeks intervention with WP pre-meals [25]. Additionally, we observed a difference in S-paracetamol concentration between the two groups at the end of the meal test. The higher S-paracetamol concentration in non-diabetics may indicate that the stomach is not empty in these subjects after 6-h. The difference between the two groups is unexpected and unexplained.

Insulin and glucagon responses were increased at 15 min (time point 0 min) and 30 min (time point 15 min) after consumption of a WP pre-meal compared with WP ingested as part of the main meal. This corroborates previous results [12,13] and underlines the insulinotropic effects of WP. A WP pre-meal stimulates insulin secretion more than when WP is part of the main meal. The significant difference between the groups during the postprandial period implies a lower response for non-diabetic than for diabetic subjects. Even though we observed significant differences in hormone secretion between the groups, there was no interaction between diabetes and intervention for any of the measured parameters. This indicates that the WP pre-meal is not more effective in subjects with than without T2D. As expected the glucose responses to the pre-meal were exaggerated in T2D subjects compared to non-diabetic subjects. However, there was no impact of the WP pre-meal in any of the two groups. The suppressive effect on glucose of 45 g protein found previously in T2D [10] differs from the present study. The reason for this discrepancy may be related to the different protein amount which causes different insulin responses. For glucagon, we observed a third-order interaction between diabetes, intervention and time with a higher glucagon concentration in the diabetic group. This suggests that mean glucagon concentration during the postprandial period is significantly dependent on both diabetic status and intervention.

We detected enhanced GIP responses to WP. Interestingly, the WP pre-meal stimulated GIP concentrations more potently than when WP was part of the fat-rich meal. It has been shown, that both fat and WP stimulate GIP secretion [26]. Hence, there may be a synergistic effect between WP and fat being more pronounced to a WP pre-meal than when WP is consumed as part of a meal in both subjects with and without T2D. GIP has been proposed to be a regulator of the lipid metabolism [27]. The GIP secretion is preserved and nearly normal in subjects with T2D [28], which may explain why there was no interaction between diabetes and time. The GLP-1 did not differ and the responses were not affected by diabetes or by the WP pre-meal. This finding contrasts previous acute and four-week studies in subjects with T2D, where a WP pre-meal significantly increased GLP-1 secretion [12,25]. The reason for the discrepancy is not known but could be related to the different doses of WP.

WP has satiating properties compared with other protein sources as well as with a pre-load of water [29,30]. However, we observed no difference between groups or between interventions for the four appetite parameters (fullness, satiety, hunger and prospective consumption). The reason may be that the huge energy density in the fat-rich main meal dominates and overshadows the appetite related feelings, thereby weakening the potential contribution of the WP pre-meal. 

Our study has some strength. Firstly, we applied a cross-over design with two groups that were matched for BMI, sex and age. Secondly, the effect of a WP pre-meal was compared with WP as part of the fat-rich meal, which allowed us to conclude that a pre-meal is more effective in stimulating hormone responses and delaying gastric emptying. Thirdly, the practical relevance makes the pre-meal concept easy to adapt e.g., to insulin-treated subjects with T2D. Additionally, we present the non-fasting responses of TG which are a more robust indicator of CVD risk than fasting TG [31]. Nevertheless, our study also has some limitations. We intended to recruit healthy controls. However, many controls matching the BMI of the T2D subjects had some degree of metabolic disturbances. Another weakness is the duration of the test period, which should ideally continue until biochemical parameters had reached a steady state. When the test was discontinued after 6 h, however, some of the metabolic parameters, e.g., lipid responses, had not yet reached basal levels. Whether a long-term study may provide different results from our acute study warrants further investigation. Administration of medication may be a restriction in the study despite no change in doses was allowed during the study. Thus lipid-lowering drugs may potentially affect the results but we adjusted our statistical analysis to account for this factor and found no influence. 

## 5. Conclusions

In conclusion, we demonstrated that a WP pre-meal before a fat-rich meal had a differential effect on hormone (insulin, glucagon and GIP) and lipid responses in both subjects with and without T2D. Hormone responses were more pronouncedly stimulated by a 20 g WP pre-meal than by 20 g WP as part of the fat-rich meal. In addition, we found that a WP pre-meal reduces gastric emptying in both groups. Apparently, a pre-meal 20 g WP did not have a clinically relevant impact on the metabolism.

## Figures and Tables

**Table 1:** Clinical characteristics. 24 subjects divided into two groups—type 2 diabetics and non-diabetics ^1^.

Clinical Characteristic, Unit	Type 2 Diabetics (*n* = 12)	Non-Diabetics (*n* = 12)	*P* Value ^2^
Gender, f/m	3/9	3/9	-
Age, years	62.9 (57.0–68.8)	62.8 (57.6–67.7)	0.944
Weight, kg	89.1 (77.3–100.8)	84.6 (78.1–91.1)	0.473
Fasting plasma glucose, mmol/L	8.77 (7.6–10.0)	5.7 (5.5–5.9)	<0.0001
HbA1c, %	49.6 (46.9–52.3)	36.5 (33.8–39.2)	<0.0001
Fasting triglycerides, mmol/L	1.76 (0.88–2.28)	1.33 (0.92–1.73)	0.502
HDL cholesterol, mmol/L	1.3 (1.1–1.5)	1.5 (1.2−1.9)	0.226

^1^ Values are means (95% confidence interval). ^2^ Groups are compared by paired *t*-test. Abbreviations: HbA1c: haemoglobin A1c, HDL: high-density lipoproteins.

**Table 2:** Postprandial concentrations (95%-CI) to selected time points of biochemical parameters during the postprandial period. Subjects with (*n* = 12) and without type 2 diabetes (T2D) (*n* = 12) ^1^ were observed for 360 min after consumption of a pre-meal of whey proteins or water before a fat-rich main meal.

		Type 2 Diabetes		Non-Diabetes		*P*-Value ^3^		
Parameter, Unit	Time, min	Whey Protein	Water	Whey Protein	Water	Diabetes × Intervention	Diabetes × Time	Intervention × Time
ApoB-48 ^2^, µg/L	−15 (fasting)	8596 (6847–10346)	8750 (6968–10532)	9141 (7304–10978)	9348 (7470–11227)	0.9282	0.0039	0.9416
	240	23340 (18591–28090)^x^	23758 (18920–28595)^x^	18068 (14438–21699)^y^	18478 (14766–22191)^y^			
	360	18029 (14360–21697)^x^	18351 (14614–22088)^x^	14202 (11349–17056)^y^	14525 (11606–17443)^y^			
Triglycerides ^2^, mmol/L	−15 (fasting)	1.47 (1.17–1.76)	1.52 (1.22–1.82)	1.25 (1.00–1.50)	1.27 (1.02–1.52)	0.9994	0.1011	0.9852
	240	2.53 (2.02–2.03)	2.62 (2.10–3.14)	2.15 (1.72–2.58)	2.19 (1.75–2.63)			
	360	2.04 (1.63–2.45)	2.11 (1.69–2.53)	1.74 (1.39–2.08)	1.77 (1.41–2.11)			
NEFA, mmol/L	−15 (fasting)	0.62 (0.58–0.66)	0.60 (0.56–0.64)	0.53 (0.49–0.57)	0.52 (0.48–0.56)	0.8925	0.7671	0.6513
	120	0.26 (0.21–0.30)	0.23 (0.19–0.28)	0.17 (0.13–0.21)	0.16 (0.11–0.20)			
	360	0.55 (0.51–0.59)	0.53 (0.49–0.57)	0.47 (0.42–0.51)	0.44 (0.41–0.49)			
Insulin ^2^, pmol/L	−15 (fasting)	60.9 (43.7–78.1)	63.1 (45.5–80.6)	50.3 (36.4–64.2)	52.9 (28.3–67.5)	0.2555	<0.0001	<0.0001
	15	266.9 (192.7–341.2)^x^	179.9 (129.9–230.0)^y^	259.9 (188.3–331.5)^x^	175.8 (127.4–224.4)^y^			
	120	218.5 (157.7–279.3)	208.4 (150.4V–266.4)	124.1 (89.9–158.2)	118.8 (86.1–151.5)			
Glucagon ^2^, pg/mL	−15 (fasting)	84.5 (76.9–92.1)	83.3 (75.8–90.8)	74.1 (67.5–80.7)	71.9 (65.5–78.4)	0.8994	0.1111	<0.0001
	15	153.2 (139.5–166.9)^x^	118.5 (107.9–129.1)^y^	134.3 (122.3–146.3)^x^	102.3 (93.2–111.5)^y^			
	120	111.1 (101.1–121.0)	117.9 (107.3–128.4)	97.4 (88.7–106.1)	101.8 (92.7–110.8)			
Glucose, mmol/L	−15 (fasting)	8.47 (8.08–8.85)	8.58 (8.19–8.96)	5.61 (5.22–5.99)	5.47 (5.08–5.9)	0.0865	<0.0001	0.5188
	30	9.83 (9.44–10.21)^x^	9.94 (9.55–10.32)^x^	5.69 (5.58–6.35)^y^	5.82 (5.44–6.21)^y^			
	120	9.85 (9.46–10.23)^x^	9.96 (9.57–10.34)^x^	5.46 (5.08–5.85)^y^	5.33 (4.94–5.71)^y^			
GLP-1 ^2^, pmol/L	−15 (fasting)	21.6 (18.6–24.5)	19.5 (16.8–22.3)	14.2 (12.4–16.0)	13.7 (11.9–15.5)	0.4138	0.8896	0.4129
	120	47.7 (41.2–54.3)	43.3 (37.2–49.3)	31.4 (27.3–35.5)	30.4 (26.4–34.3)			
	360	26.0 (22.4–29.6)	23.6 (20.2–26.9)	17.1 (14.9–19.3)	16.5 (14.4–18.7)			
GIP ^2^, pmol/L	−15 (fasting)	9.7 (7.9–11.5)	10.7 (8.8–12.7)	8.5 (7.0–10.1)	9.7 (7.9–11.5)	0.9829	0.2080	<0.0001
	120	60.9 (49.7–72.1)^x^	51.6 (42.1–61.1)^y^	53.7 (43.9–63.6)^x^	46.6 (38.0–55.2)^y^			
	360	28.8 (23.4–34.0)	27.7 (22.6–32.8)	25.3 (20.7–30.0)	25.0 (20.4–29.6)			
S-paracetamol ^2,4^, µmol/L	30	53.5 (33.8–73.2)^x^	107.8 (67.0–148.6)^y^	55.8 (35.3–76.3)^x^	116.4 (72.3–160.4)^y^	0.4115	0.0271	0.0390
	120	56.9 (36.1–77.7)	58.7 (37.4–80.2)	60.0 (38.1–81.9)	64.2 (40.75–9.6)			

^1^ Values are means (95% confidence interval) unless otherwise stated. ^2^ Medians (95% confidence interval). ^3^ The hypothesis was to test if the response curves for the two interventions during the postprandial period were parallel. No third-order interactions were found for the measured parameters except for glucagon (*P* = 0.0340). ^4^ All fasting values below detection limit. Values in a row with different superscript letters are significantly different, *P* < 0.05. Abbreviations: ApoB-48: apolipoproteins B-48, NEFA: non-esterified fatty acids, GLP-1: glucagon-like polypeptide 1, GIP: gastric inhibitory polypeptide, WP: whey proteins.

## Supplementary Materials

The following are available online at http://www.mdpi.com/2072-6643/10/2/122/s1, Table S1: Means (95%-CI) of appetite parameters. Subjects with (*n* = 12) and without type 2 diabetes (T2D) (*n* = 12) were observed after consumption of a pre-meal of whey proteins (WP) or water followed by a fat-rich main meal.

## Abbreviations

ApoB-48	Apolipoprotein B-48
DPP-4	Dipeptidyl peptidase 4
EDTA	Ethylene-diamine-tetra acetic acid
ELISA	Enzyme-linked immunosorbent assay
E%	Energy percentage
GIP	Glucose-dependent insulinotropic peptide
GLP-1	Glucagon-like peptide-1
HbA1c	Haemoglobin A1c
iAUC	Incremental area under the curve
MUFA	Monounsaturated fatty acids
NEFA	Non-esterified fatty acids
PPL	Postprandial hypertriglyceridemia
PUFA	Polyunsaturated fatty acids
SEM	Standard error of the mean
SFA	Saturated fatty acids
TG	Triglyceride
T2D	Type 2 diabetes
VAS	Visual analogue scale
WP	Whey proteins
