# Supplementary material for: Pre-Meal Effect of Whey Proteins on Metabolic Parameters in Subjects with and without Type 2 Diabetes: A Randomized, Crossover Trial
Source: Nutrients. 2018 Jan 25;10(2):122. doi: 10.3390/nu10020122 (PMC5852698; doi:10.3390/nu10020122)
Supplement: Supplementary file 1 [file nutrients-10-00122-s001.pdf]

Supplementary Table S1. Means (95%-CI) of appetite parameters. Subjects with (*n* = 12) and without type 2 diabetes (T2D) (*n* = 12) [1] were observed after consumption of a pre-meal of whey proteins (WP) or water followed by a fat-rich main meal.

	**Type 2 diabetes**		**Non-diabetes**		**ANOVA, *P*-Value [3]**		
**Parameter**	**Whey protein**	**Water**	**Whey protein**	**Water**	**Diabetes × intervention**	**Diabetes × time**	**Intervention × time**
Fullness, mm	9 457 (5 829–13 085)	14 169 (10 540–17 797)	14 052 (10 424–17 681)	11 448 (7 820–15 076)	0.7553	0.7859	0.6666
Satiety, mm	11 384 (8 307–14 462)	10 363 (7 286–13 441)	10 912 (7 835–13 989)	10 876 (7 798–13 953)	0.6124	0.8530	0.8836
Hunger[2], mm	12 070 (8 677–15 463)	9 978 (6 585–13 371)	9 245 (5 852–12 639)	7 501 (4 108–10 894)	0.4170	0.9778	0.1897
Prospective[2], mm	7 930 (5 022–10 837)	8 388 (5 481–11 295)	8 899 (5 992–11 806)	9 243 (6 336–12 150)	0.2542	0.8482	0.7501

[1] Values are means (95% confidence interval) unless otherwise stated. [2] Medians (95% confidence interval). [3] The hypothesis was to test if the response curves for the two interventions during the postprandial period were parallel.No third-order interactions were found for the measured parameters except for glucagon (*P* = 0.0352). Significant main effects were found for diabetes on perspective consumption (*P* = 0.0216) and of intervention on fullness (*P* = 0.0135). No significant main effects of diabetes on fullness (*P* = 0.2765), satiety (*P* = 0.1803) and hunger (*P* = 0.1526). No significant main interaction of intervention satiety (*P* = 0.1506), hunger (*P* = 0.436) and prospective consumption (*P* = 0.463).
